# Prenatal Tobacco Exposure Modulated the Association of Genetic variants with Diagnosed ADHD and its symptom domain in children: A Community Based Case–Control Study

**DOI:** 10.1038/s41598-019-40850-w

**Published:** 2019-03-12

**Authors:** Yanni Wang, Dan Hu, Wenjing Chen, Hongli Xue, Yukai Du

**Affiliations:** 1 Department of Maternal and Child Health, School of Public Health, Tongji Medical College, Huazhong University of Science and Technology, Wuhan, Hubei P. R. China; 2 Department of Child Health Care, Hospital of Maternal and Child Health of Dalian, Dalian, Liaoning P. R. China; 3 Department of Maternal, Child and Adolescent Health, School of Public Health, Lanzhou University, Lanzhou, Gansu P. R. China

## Abstract

The purpose of our study was to test the hypothesis that prenatal tobacco smoking exposure (PSE) could modulate the association of genetic variants with ADHD. A community based case-control study was conducted among Chinese children and 168 ADHD patients and 233 controls were recruited by using combination diagnosis of DSM-IV, SNAP-IV and semi-structured clinical interview. Logistic regression analysis was performed to estimate the effect of prenatal tobacco smoking exposure and genotype frequencies on ADHD susceptibility individually by adjustment for potential confounders. Multiplicative and additive interaction analysis were performed to evaluate the interactions between risk genes and PSE with regard to ADHD. Prenatal tobacco smoke exposure was a significant risk factor of ADHD even after adjusted for other potential confounders. *ADRA2A* rs553668, *DRD2* rs1124491 and *SLC6A4* rs6354 were identified to be associated with ADHD. A significant multiplicative and additive gene-environment interactions were observed between the PSE and the *ADRA2A* rs553668 in relation to ADHD and ADHD-ODD. The risk of the genetic variants in ADHD was increased significantly if the child had prenatal tobacco exposure. The genetic risk for ADHD could be influenced by the presence of environmental risks. The environmental and the genetic risks are not distinct to each other. More gene-environment interaction studies were needed to reveal the etiology of ADHD.

## Introduction

Attention-deficit/hyperactivity disorder (ADHD) is a chronic neurodevelopmental disorder characterized by developmentally inappropriate level of inattentiveness and hyperactivity. It is one of the most frequently diagnosed disorders among children and adolescents, with the pooled prevalence of 7.2%^1^ worldwide and 6.26%^2^ in China according to the most recent meta-analysis. Previous studies reveal that individuals affected by ADHD frequently lead to various social functional impairment, such as social communication disorder, bad peer relationships, academic performance difficulties^3^, develop personality disorder, substance abuse and even criminality^4,5^.

As a complex multi-factorial disease, exact etiology of ADHD is still elusive. The biologic basis for ADHD is thought to be largely genetic^6,7^. The genetic architecture of ADHD is complex and numerous single nucleotide polymorphisms (SNPs) of genes involved in the regulation of neurotransmitter systems are identified to be associated with ADHD^8–10^.Among them, genes involved in the dopaminergic, serotonergic and noradrenergic pathways are the most common candidate genes for ADHD studies^7,11–13^.

Adding to genetic influence, environmental factors are assumed to be co-involved in the etiology of ADHD^14^. Some prenatal and perinatal factors are reported to be associated with ADHD, such as younger maternal pregnant age^15^, caesarean, induced delivery, preterm birth, low birth weight^16^, and mother smoking during pregnancy. With regard to prenatal smoking, it is constantly identified to be a risk factor for offspring hyperactivity symptom and ADHD^14,17–19^. Evidence from epidemiological researches suggests that maternal cigarette smoking and environmental tobacco exposure during pregnancy are all in association with high risk of ADHD. Hyunjoo Joo^20^ and his colleagues have described that independent and combined exposure to pre- and postnatal secondhand smoke were associated with ADHD, particularly with the hyperactivity symptom domain. A large population-based birth cohort have provided that maternal smoking during pregnancy was associated with children hyperactivity symptom^19^. Although large numbers of studies demonstrated a relationship between maternal smoking exposure during pregnancy and ADHD, most of previous studies have investigated the effect of smoking exposure only and there were few studies on gene–environment interactions which were more likely to play the real role in the etiology of ADHD^21^.

Importantly, the environmental risk is not distinct to the genetic influence^21^, but it may be required to uncover the genetic liability^14^. The studies about the G ene× Environment studies of gene and smoking exposure on ADHD are limited. A gene-environment interplay study reports that the co-exist group of prenatal smoking exposure and 10 R/10 R genotype of *DAT* gene showed a higher mean scores of hyperactivity-impulsivity and inattentiveness than other groups^22^. But the findings remain speculative since the participants are lack of a clinical diagnosis of ADHD, and the ADHD scores of the participants was derived from the Conners’ Parent Rating Scale which will lead to the overdiagnosis of the ADHD.

Developing of novel technologies have made it more common in identifying genetic risk factors of ADHD, however, proved the genetic architecture of ADHD to be far more complex, since the genetic influences expressed only additional risks to disease susceptibility^23^. Therefore, gene and environment interaction research will be an inevitable way to explore the etiologic mechanisms of ADHD. However, studies about the joint effects of gene and environmental factors on ADHD is limited^14^. To fill the knowledge gap, we conducted the case-control study to elucidate the potential synergistic effect of the prenatal tobacco exposure during pregnancy and children genetic variants on children diagnosed ADHD and its symptom domains.

## Results

### Characteristics of participants

The study involved 168 ADHD patients and 233 controls. A higher proportion of males was noted with the 5.2:1 boys/girls ratio. Compared with the controls, ADHD children were more likely to have younger mother (*p* < 0.05). There were no significant difference between ADHD patients and controls in terms of family history of neural system diseases, parental marital status, preterm birth (pregnancy < 37weeks), Low birth weight(birth weight <2500 g) and delivery mode, levels of blood lead and postnatal tobacco exposure. Meanwhile, ADHD children scored higher of SNAP-IV scales (Inattention domain, hyperactive domain and ODD domain) and of CBCL total scores than controls (Table 1).

**Table 1:** Characteristics of ADHD children and healthy controls.

	ADHD	Controls	*p-*value
Gender, *N*(%)			0.200
Male	141(83.93)	183(78.54)	
Female	27(16.07)	50(21.46)	
Age(year), Mean (SD)	8.55(1.62)	8.60(1.76)	0.760
Family history of neural system diseases, *N*(%)			0.570
Yes	2(1.19)	1(0.43)	
No and Unknown	166(98.81)	232(99.57)	
Parental marital status, *N*(%)			0.245
Marries	148(88.10)	214(91.85)	
Single, separated, divorced or widowed	16(11.90)	14(8.15)	
Pregnancy age of mother(y), mean(S.D.)	26.54(4.19)	27.57(5.11)	**0**.**028**
Low birth weight(<2500 g), *N*(%)	12(7.14)	17(7.30)	0.953
Preterm birth(<37 weeks), *N*(%)	17(10.12)	21(9.01)	0.695
Delivery mode, *N*(%)			0.254
Cesarean section	50(29.76)	57(24.47)	
Vaginal delivery	118(70.24)	176(75.54)	
Breast feeding, *N*(%)	101(60.11)	146(62.66)	0.161
Intelligence Quotient, mean (S.D.)	98.50(12.07)	98.90(13.02)	0.730
SNAP-IV rating scales, mean(S.D)			
Inattention scores	1.95(0.49)	0.64(0.42)	**<0**.**001**
Hyperactivity scores	1.40(0.73)	0.42(0.36)	**<0**.**001**
Oppositional defiant scores(ODD)	1.04(0.69)	0.45(0.42)	**<0**.**001**
CBCL scales, mean(S.D)	44.87(25.07)	18.91(18.89)	**<0**.**001**
Blood lead (ug/dL), mean (S.D.)	3.58(1.59)	3.26(1.69)	0.846
Male	3.65(1.61)	3.57(1.79)	0.345
Female	3.64(1.17)	3.63(1.16)	0.722
Inattention	3.45(1.58)	3.36(1.69)	0.345
Hyperactivity/impulsivity	3.64(1.58)	3.63(1.71)	0.721
ADHD-ODD(N = 40)	3.71(1.59)	3.63(1.70)	0.111
Prenatal tobacco exposure, *N*(%)	58(34.52)	53(22.74)	**0**.**010**
Postnatal tobacco exposure, *N*(%)	53(31.54)	55(23.61)	0.087
Drinking during pregnancy, *N*(%)	4(2.4)	6(2.6)	0.896

*p*-value was calculated using chi-squared test if percentages are compared, or *t* test if means are compared.

### Associations between the *ADRA2A*, *DRD2*, and *SLC6A4* genetic variants and ADHD susceptibility

A total of 22 SNPs were detected. The genotype distributions of *SLC6A4* rs25533 and rs2020923, and *SLC6A3* rs2975226 in the controls deviated from Hardy–Weinberg equilibrium (P < 0.05). Thus they were excluded from further analysis. Consequently, a total of 19 SNPs remained in the subsequent analysis

In individual SNP association analysis, *ADRA2A* rs553668, *DRD2* rs1124491 and *SLC6A4* rs6354 were nominally significant at the uncorrected 0.05 significance level with adjustment for age and gender. Among these SNPs, *ADRA2A* rs553668G allele was associated with an increased risk of ADHD in the additive model, and *SLC6A4* rs6354 (GG + GT) was associated with an increased risk of ADHD, whereas *DRD2* rs1124491 (AA) was associated with a decreased risk of ADHD. After FDR adjustment, *SLC6A4* rs6354 remained statistically significant (*P* = 0.019, Table S1).

As the Table 2 illuminated, the detected SNPs were not associated with all symptom domains of ADHD. After adjusting for age and gender, individuals carrying the *ADRA2A* rs553668 GG or AG genotype showed an increased risk of ADHD compared with those carrying AA genotype (adjusted OR = 1.76, 95% CI = 1.12–2.77, *P* = 0.013), but the SNP was not associated with ADHD-ODD. Additionally, individuals who carried *DRD2* rs1124491 AG or GG genotype displayed a risk association with inattention symptom compared to AA genotype (adjusted OR = 2.22, 95% CI = 1.11–4.46, *P* = 0.022). Compared with those carrying TT genotype, the risk association with ADHD-ODD was increased significantly in individuals carrying the *SLC6A4* rs6354 GT or GG genotype (adjusted OR = 2.18, 95% CI = 1.04–4.56, *P* = 0.034), but no association was observed in other ADHD symptom domains. The ORs and 95% CIs changed slightly in different adjustment model (Model 1, 2, 3, Table 2).

**Table 2:** Association Between risk genotypes of *ADRA2A*, *DRD2* and *SLC6A4* genes and ADHD with Symptom Domains.

Symptom domain(N)	Model 1		Model 2		Model 3	
	OR	(95% CI)	OR	(95% CI)	OR	(95% CI)
*ADRA2A* rs553668^a^						
All ADHD(160)	1.76^*^	(1.12–2.77)	2.07^*^	(1.27–3.37)	2.00^*^	(1.23–3.23)
Inattention(141)^d^	1.91^*^	(1.19–3.07)	2.05^*^	(1.26–3.36)	2.19^*^	(1.32–3.63)
Hyperactivity/impulsivity(78)^e^	2.18^*^	(1.21–3.94)	2.28^*^	(1.24–4.18)	2.37^*^	(1.29–4.34)
ODD(39)^f^	1.53	(0.72–3.25)	1.79	(0.82–3.91)	2.07	(0.92–4.66)
Control(225)	1		1		1	
*DRD2* rs1124491^b^						
All ADHD(163)	2.22^†^	(1.11–4.46)	2.11^†^	(1.01–4.06)	2.38^†^	(1.33–5.83)
Inattention(143)^d^	2.11^†^	(1.01–4.06)	2.42^†^	(1.15–5.11)	2.11^†^	(1.03–4.39)
Hyperactivity/impulsivity(78)^e^	1.73	(0.76–3.94)	1.83	(0.79–4.26)	1.81	(0.78–4.19)
ODD(36)^f^	1.58	(0.52–4.77)	1.78	(0.57–5.59)	1.63	(0.53–5.00)
Control(222)	1		1		1	
*SLC6A4* rs6354^c^						
All ADHD(167)	1.73^†^	(1.07–2.78)	1.51	(0.91–2.51)	1.55	(0.93–2.59)
Inattention(153) ^d^	1.58	(0.97–2.56)	1.40	(0.84–2.42)	1.42	(0.83–2.43)
Hyperactivity/impulsivity(85)^e^	1.62	(0.91–2.88)	1.56	(0.85–2.84)	1.48	(0.80–2.73)
ODD(39)^f^	2.18^†^	(1.04–4.56)	2.38^†^	(1.09–5.19)	2.46^†^	(1.10–5.50)
Control(222)	1		1		1	

^a^The ORs and 95% CIs were estimated the GG/AG vs AA genotype of *ADRA2A* rs553668; ^b^The ORs and 95% CIs were estimated the GG/AG vs AA genotype of *DRD2* rs1124491: ^c^The ORs and 95% CIs were estimated the GT/GG vs TT genotype of *SLC6A4* rs6354.d: all the patients with SNAP-IV inattention subscale score ≥ 1.6.e: all the patients with SNAP-IV hyperactivity subscale score ≥ 1.6.f: all the ADHD patients with SNAP-IV ODD subscale score ≥ 1.6.

The ORs and 95% CIs were estimated by logistic regression model. Model 1:adjusted for participants age and gender; Model 2: variables in model 1plus pregnancy age of mother, family history of nervous system diseases, low birth weight, preterm birth; Model 3, variables in model 2 plus the blood lead concentration and postnatal tobacco smoke exposure.

^*^p < 0 0.01, ^†^*p* < 0.05.

### Prenatal tobacco smoke exposure (PSE) as a risk factor for ADHD

The prenatal tobacco smoke exposure (PSE) showed a risk association with ADHD and all symptom domains, and slight change of the OR and 95% CI was observed in different adjustment model (Model1, 2, 3). The strong association was shown in ODD symptom domain (OR = 2.80, 95% CI = 1.39–5.66) and in the hyperactivity symptom domain (OR = 2.12, 95% CI = 1.23–3.60, Table 3).

**Table 3:** Association between the prenatal tobacco smoke exposure and ADHD with the symptom domains.

Symptom domain(*N*)	Model 1		Model 2		Model 3	
	OR	(95% CI)	OR	(95% CI)	OR	(95% CI)
All ADHD(*N* = 168)	1.77^a^	(1.14–2.76)	1.90^a^	(1.19–3.05)	1.75^b^	(1.07–2.94)
Inattention(*N* = 154)^a^	1.76^b^	(1.16–2.77)	1.88^b^	(1.16–3.04)	1.71^b^	(1.01–2.90)
Hyperactivity(*N* = 86)^b^	2.12^a^	(1.23–3.60)	2.29^a^	(1.31–4.00)	2.34^a^	(1.23–4.47)
ODD(*N* = 40)^c^	2.80^a^	(1.39–5.66)	2.94^a^	(1.41–6.12)	3.15^a^	(1.38–7.21)
Control(*N* = 233)	1		1		1	

^a^all the patients with SNAP-IV inattention subscale score ≥ 1.6. ^b^all the patients with SNAP-IV hyperactivity subscale score ≥ 1.6. ^c^all the ADHD patients with SNAP-IV ODD subscale score ≥ 1.6.

The ORs and 95% CIs were estimated for PSE using logistic regression model. Model 1: adjusted for participants age and gender; Model 2: variables in model 1plus pregnancy age of mother, family history of nervous system diseases, low birth weight, preterm birth; Model 3, variables in model 2 plus the blood lead concentration and postnatal tobacco smoke exposure.

^a^*p < *0.01,^b^*p* < 0.05.

### Interaction between prenatal tobacco smoking exposure and genetic variants

The MDR analysis revealed that the three-factor model including rs553668, rs1124491 and prenatal tobacco smoking exposure was the best predictor for ADHD risk (Table 4).

**Table 4:** MDR analyses of the gene-environment interactions between SNP rs553668, rs1124491, rs6354and risk factors in ADHD risk.

Model	TBA	CV consistency	P value
*ADRA2A* rs553668	0.5215	8/10	0.018
*ADRA2A* rs553668, *DRD2* rs1124491	0.5946	9/10	0.000
*ADRA2A* rs553668, *DRD2* rs1124491,PSE	0.6310	10/10	0.000
*ADRA2A* rs553668,PSE	0.6120	8/10	0.000

PSE: prenatal tobacco smoking exposure, CV consistency, cross-validation consistency. TBA: testing balance accuracy.

Furthermore, we calculated the multiplicative and additive interactions between *ADRA2A* rs553668, *DRD2* rs1124491, *SLC6A4* rs6354 and prenatal tobacco smoking exposure after adjustment of age of the child, gender, pregnancy age of mother, and postnatal smoking exposure.

A significant additive interaction between PSE and *ADRA2A* rs553668 polymorphisms is detected. The AP due to interaction was 0.48(95% CI = 0.09–0.87), and RERI was 1.88(95% CI = 0.06–4.12), Meanwhile, multiplicative interaction was displayed in this group (*P*_mul_ = 0.003). The multiplicative and additive interactions were found in all symptom domain of ADHD. However, no multiplicative and additive interactions were detected between *DRD2* rs1124491 polymorphism and PSE, nor between *SLC6A4* rs6354 polymorphism and PSE (Table 5).

**Table 5:** Interactions between PSE and genetic variants on child ADHD risk

PSE	Genotype	Cases, *N*(%)	Controls, *N*(%)	OR(95% CI)	*P*_mul_	AP(95% CI)	RERI(95% CI)
***ADRA2A*** **rs553668**							
No	AA	26(16.25)	61(27.11)	1	0.003	0.48(0.09–0.87)	1.88(0.06–4.12)
Yes	AA	14(8.75)	23(10.22)	1.36(0.58–3.22)			
No	AG + GG	76(47.50)	115(51.11)	1.65(0.93–2.90)			
Yes	AG + GG	44(27.50)	26(11.56)	3.54(1.73–7.25)			
***DRD2*** **rs1124491**							
No	AA	7(4.27)	27(12.16)	1	0.175	−0.55(−1.84–0.73)	−3.34(−11.42–4.74)
Yes	AA	99(60.36)	145(65.32)	5.34(1.64–4.46)			
No	AG + GG	6(3.66)	6(2.70)	3.99(1.46–0.89)			
Yes	AG + GG	52(31.71)	44(19.82)	6.04(2.07–7.65)			
***SLC6A4*** **rs6354**							
No	TT	74(44.31)	147(63.91)	1	0.234	−0.80(−2.42–0.82)	−1.37(−3.39–0.65)
Yes	TT	44(26.35)	39(16.96)	2.13(1.20–3.78)			
No	TG + GG	34(20.36)	32(13.91)	1.97(1.10–3.52)			
Yes	TG + GG	15(8.98)	12(5.22)	1.79(0.75–4.28)			

*P*_mul_ was calculated using the multiplicative interaction term in the logistic regression analysis.

The ORs, 95% CIs, AP and RERI were adjusted by age, gender, pregnancy age of mother, and postnatal smoking exposure.

**Figure 1:**
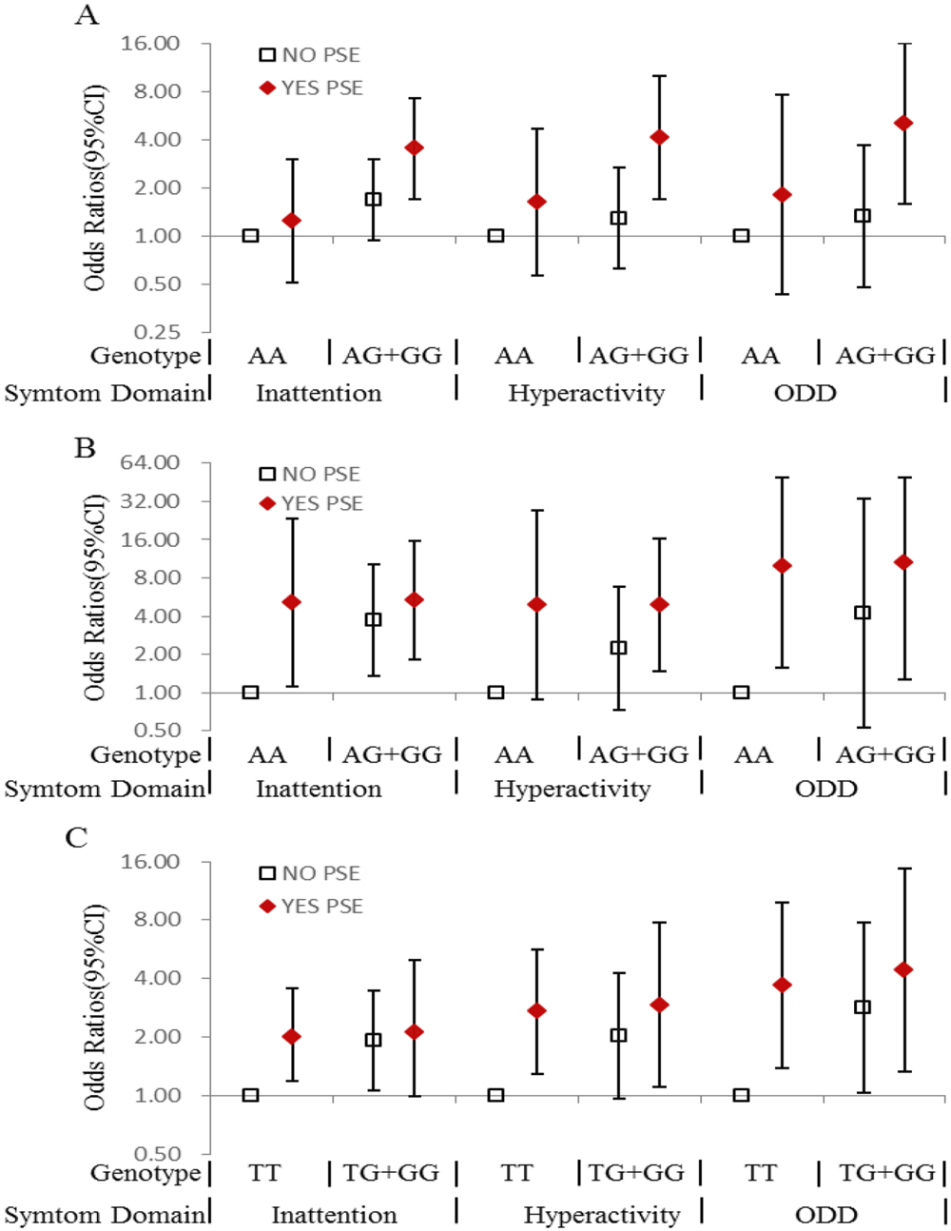
Prenatal tobacco exposure and genetic variant in association with ADHD and its symptom domains. (**A**) *ADRA2A* gene (rs553668) variant and prenatal tobacco exposure in association with ADHD and its symptom domains; (**B**) *DRD2* gene (rs1124491) and prenatal tobacco exposure in association with ADHD and its symptom domains: (**C**) *SLC6A4* gene (rs6354) and prenatal tobacco exposure in association with ADHD and its symptom domains.

Figure 1 described the ORs and the 95% CIs for the only unfavorable genotype exposure, the only prenatal tobacco smoking exposure and the joint effect of them with regard to ADHD and all symptom domains. Most of the risk genotypes showed an increased risk association with ADHD when co-existed with PSE compared with individuals without PSE. The risk association of ADHD in *ADRA2A* rs553668 GG or AG carrier without PSE was moderately increased (OR = 1.65, 95% CI = 0.93–2.90, *P* = 0.084), compared with AA carrier without PSE. But the OR for the rs553668 GG or AG carriers with PSE, was significantly increased to 3.54(95% CI: 1.73–7.25, *P* = 0.001 Table S2). The group of *DRD2* rs1124491 AG/GG genotype with PSE also showed a significant association with ADHD compare with whose without PSE (OR = 6.04, 95% CI = 2.07–17.65, *P* = 0.001, Table S3). And *SLC6A4* rs6354 GT/GG genotype carriers with PSE also showed an increased association in hyperactivity and ADHD-ODD symptoms (hyperactivity domain: OR = 2.92, 95% CI = 1.10–7.75, *p* = 0.031; ODD domain: OR = 4.42 95% CI = 1.32–14.79, *p* = 0.016.Table S4).

## Discussion

The current case-control study demonstrated a significantly association of prenatal tobacco exposure with all ADHD subtype and ADHD concurring with ODD(ADHD-ODD) in children, especially a strong association with hyperactivity and ADHD-ODD, and the effect size did not diminished even after adjusted by potential environmental influencing covariates. In addition, the study found three SNPs of *ADRA2A*, *DRD2* and *SLC6A4* genes associated with ADHD, but the association disappeared in some ADHD subtypes after adjusted by potential environmental covariates. Interestingly, when grouped with prenatal tobacco exposure, however, the association of the genetic variants and ADHD reappeared although there are no multiplicative and additive interaction between *DRD2*, *SLC6A4* genes and PSE.

With regard to the findings in the genetic analysis, the results of risk association with ADHD in the children carrying *ADRA2A* rs553668 GG or AG genotype were in concordance with the findings of several studies, and indicated that rs553668, being known as the DraI site in the 3′-untranslated region of *ADRA2A* gene, A-to-G variant increased the risk of ADHD^24,25^. Meanwhile, the findings showed the *DRD2* rs1124491 in the significant association with inattention type of ADHD. *DRD2* gene (Dopamine Receptor D2 gene), encodes the D2 subtype of the dopamine receptor. A population-based birth cohort have reported an association of *DRD2* rs1124491 with ADHD in males and low persistence in female ADHD patients^26,27^. Moreover, a common variant *SLC6A4* rs6354 was identified to be in association with ADHD-ODD. *SLC6A4* rs6354 located in the untranslated(exon 2) 5′-UTR region of *SLC6A4* gene—a potential module of exonic splicing enhancer or silencer by bioinformatics prediction— and may affect the expression level or exonic splicing of serotonin^28^. Evidences show that one repeat length polymorphism-STin2 located in the untranslated exon 2 of *SLC6A4* gene may be significantly associated with major depressive disorder and suicide^29^.

A limited number of G × E studies have shown that genetic factors interact with maternal smoking to heighten risk for ADHD. Anita Thapar^30^ and his colleagues found maternal smoking during pregnancy was shown a significant association with offspring ADHD symptom scores in twins study after adjustment by genetic factors, although the main effect of cigarette exposure was small. One gene-environment interaction study evaluated maternal smoking and the *DAT* gene. In the cases with co-exist of maternal smoking during pregnancy and 10 R/10 R genotype of *DAT* gene, the mean score of hyperactivity-impulsivity and inattentiveness derived from the Conners’ Parent Rating Scale were higher than other groups^22^. The findings in the current study infer that the susceptibility of ADHD for the unfavorable ADHD genotypes carriers might increase when the children having the history of in-utero exposure to cigarette smoking.

However, the molecular mechanisms responsible for the interaction remain unknown. Nicotine, the main psychoactive component of tobacco, can accumulated in human brain fast when they are smoking^31^. The neurotoxic effects of nicotine exposure on human fetal brain development have not been studied directly. However, a large number of animal studies have found that nicotine has obvious neurotoxic effects on fetal brain development^32^. Animal models confident that the nicotine can readily cross the placental barrier into fetal circulation, and the blood-brain barrier, which protects the adult brain from toxic chemicals, is not fully formed until about six months after birth^33^. So, nicotine can cross the blood-brain barrier and specifically binds to nicotinic acetylcholine receptors (nAChRs) in the fetal brain^34^, and nAChRs have an important role in regulating synaptic plasticity and brain development. Concerning of animal models, chronic nicotine exposure to animal brain developmentally leads to long-term changes of decreasing presynaptic nAChRs function, which cause altering nicotine-stimulated release of those neurotransmitters and the altering continues for a long time even after nicotine exposure has been removed^35,36^. And neurotransmitters dysregulation of acetylcholine, dopamine and serotonin are assumed to be the reason of ADHD^21^. The decreased presynaptic nAChRs function may increase the risks of dysfunctions of dopaminergic, serotonergic and noradrenergic neurotransmission caused by genetic variants. That may explain the strong joint effects of prenatal tobacco exposure and genetic variants on all ADHD subtypes. But the exact reasons of the increasing risks require more extensive research.

Lead is a heavy metal that exists in the environment in nature, and it is a most widely used neurotoxicants. Many studies have shown that high blood lead levels (≥10 µg/dL) are harmful to children neurodevelopment^37,38^. Moreover, evidences support that longer term low level exposure (<10 µg/dL) associated with intellectual impairment^39,40^. Unfortunately, the effects of lead exposure on ADHD are less clear. In the current study, no statistically significant association was found between low blood lead levels (<10 µg/dL) and diagnosed ADHD and all the symptoms. A growing body of evidence now support that low level exposure (<5 µg/dL) also associated with cognitive impairment, suggesting that there is no threshold for developmental neurotoxicity^37,41,42^. Stephani Kim^5^ and his colleagues examined the relationship between blood lead levels and ADHD in children, and found that the odds ratios for the risk of ADHD is 4.63 (95% CI: 1.36–15.72) in the “≥2 mg/dL” group and the odds ratios increased to 7.25 (1.66–31.67) in the “≥3 mg/dL” group. However, some studies reported that the association between blood lead levels and ADHD did not exhibit a dose-response relationship^43^.

To the best of our knowledge, this paper is the first G × E interaction research about SNPs and prenatal tobacco smoke exposure in association to ADHD and all the subtypes. The major strength of this study is the standardized procedures of ADHD assessment in children and the precise and rigorous diagnosis^21^, aiming to minimize the overdiagnosis or underdiagnosis. However, several limitations have to be considered during interpretation of the findings. The major limitation of this study is that the small sample size has decreased the power to detect the effects of the SNPs and the gene-environment interactions. One inherent methodologic problem in this case-control design is that the retrospective assessment of PSE might introduce systematic error in the calculation of the association between PSE and ADHD because of recall bias. In order to diminish the recall bias, we recruited the new diagnosed patients from the community survey. Although the regression model controlled for important confounding variables such as age, gender, mother and father education, and postnatal smoke exposure status, it did not take into account other possible environmental pollutants, such as manganese, mercury, arsenic and bisphenol A and phthalates^5,44,45^, which can be a focus of future research. Psychological and social environment are also the important for child psychological development, but those did not measured in our study. This is a weakness of the study. The participants in our study were enrolled from one city of China, which limited the results interpretation in general populations. Finally, its cross-sectional nature limits explication about the causal effects of G × E interactions. Future research may focus on a longitudinal study to confirm the causality.

## Methods

### Participants

This study was approved by the Ethics Committee of Tongji Medical College, Huazhong University of Science and Technology, and all methods were performed in accordance with the approved guidelines and regulations. Informed consent was obtained from all participants and their parents.

Children who were diagnosed for ADHD by pediatric psychiatrists were recruited as the cases in the Liuzhou Women and Children Healthcare Hospital, and 6–12-year-old healthy children from 2 elementary schools in Liuzhou City were also recruited as the controls. The exclusion criteria for both groups were: 1) a history of mental retardation, autism spectrum disorders, bipolar disorder, language disorders or learning disabilities, pervasive developmental disorder; 2) a history of brain disease, seizure disorder, or other neurological disorders; 3) intelligence quotient (IQ) < 70; and 4) the presence of any chronic physical disease; 5) a blood lead concentration ≥10(µg/dL).

Permissions were given for the eligible patients and controls to participate in the study, and those children whose parents could not be contacted or denied consent for participation were excluded. As a result, a total of 168 cases and 233 controls (6–12 years of age) were recruited.

### Assessments of children ADHD

The status of both the case and control subjects were confirmed with a semi-structured clinical interview with the parents and children that was conducted according to the DSM-IV ADHD Rating Scale by a pediatric psychiatrist. Additionally, diagnoses were made that included both in categories and severity ratings using the 26-item Chinese version of SNAP-IV scale^46^. The 4-point response is scored 0–3 (Not at All = 0, Just A Little = 1, Quite a Bit = 2, and Very Much = 3) on SNAP-IV scale. Subscale scores are calculated by summing the scores on the items in the specific symptom domains (inattention, hyperactive and ODD), in which average score 0–1 means no features, 1.1–1.5 means subclinical, 1.6–2.0 means significant, and >2.1 means very severe. We defined oppositional defiant disorder as the ODD symptom domain score ≥1.6. The Child Behavior Checklist (CBCL) scale was conducted to evaluate child potential behavior problems and the Wechsler Intelligence Scale for Children (WISC) was used to evaluate full-scale IQ of all participants. All instruments used in the current study are validated Chinese versions.

### Measurement of blood lead concentrations

To measure lead concentrations in blood, 2–3 mL of whole blood was drawn from each child and kept it in heparin-containing tubes. Blood lead level in whole blood was measured using graphite furnace atomic absorption spectrometry (Spectral AA-240FS, Varian, American). The limit of detection for blood lead was 0.1 µg/dL. None of the blood samples showed below the limit of detection.

### DNA extraction and genotyping

Genomic DNA was extracted from peripheral blood leukocytes using the TIANamp® Blood DNA Kit DP318 (TIANGEN BIOTECH CO., LTD., Beijing, China).

The candidate genes within the dopamine, norepinephrine and serotonin neurotransmitter pathways were selected based on recent findings^47^, and included 7 genes (i.e., *SLC6A3*, *SLC6A4*, *DRD2*, *ANKK1*, *DRD4*, *ADRA2A*, *SNAP25*). The screening procedure for the candidate single nucleotide polymorphism(SNP) was as follow: First, we searched the F-SNP database (http://compbio.cs.queensu.ca/F-SNP) to retrieve information about the predictive functions of the SNPs within these genes and those of potential effects on splicing, transcription, translation, and post translation processes were selected. Second, we filtered the SNPs by the MAF of CHB (minor allele frequency for Han Chinese in Beijing) in the database of 1000 genomes (https://www.ncbi.nlm.nih.gov/variation/tools/1000genomes/) and selected SNPs with MAFs > 5% for the next step. Third, we tested the linkage disequilibrium (LD) among selected SNPs using SNAP Pairwise LD (http://www.broadinstitute.org/mpg/snap/ldsearchpw.php), and only one was reserved if several SNPs were in strong LD with each other (*r*^2^ ≥ 0.80). Consequently, a total of 16 SNPs were retained. High-quality genotyping was performed on the Sequenom MassARRAY platform (San Diego, USA) by BIO MIAO BIOLOGICAL TECHNNOLOGY (Beijing, China) following the standard experimental procedures from the manufacturer. In addition, samples with a call rate <80% or Hardy-Weinberg equilibrium (HWE) < 0.05 in the control group were removed.

### Determination of tobacco exposure

Tobacco exposure status was obtained from the children’s parents by a questionnaire. The questions were constructed in two parts: mother tobacco exposure and child secondhand smoke exposure. Firstly, we asked whether the mother smoked before pregnancy or not (>100 cigarettes) and whether she smoked during pregnancy. If yes, we then asked how many cigarette expensed per day. Only three of the mothers reported being an ex-smoker or having smoked during pregnancy. Besides, we inquired whether or not she was exposed to secondhand smoke during pregnancy at home or in the workplace and how long she stayed in the smoking circumstance per day/week. Secondly, we investigated whether any family members smoked at home after the child birth and how many cigarette the members expensed per day/week.

### Confounders and Covariates

A face-to-face questionnaire survey was administered for their parents by a trained interviewer to collect socio-demographic variables, prenatal and perinatal characteristics. Socio-demographic variables included age, gender, and parental marital status (categorized as married, or single, separated, divorced and widowed). Prenatal and perinatal characteristics included birth weight, pregnancy age of mother, preterm birth and the mode of delivery (cesarean section or vaginal delivery). The family history of neural system diseases and ADHD were asked additionally, but no participant declared of ADHD family history in their families.

### Statistical Analysis

Differentials in the distribution of socio-demographic variables, prenatal and perinatal characteristics between patients and controls were compared by chi-squared test or *t* test, when appropriate. Hardy-Weinberg equilibrium (HWE) for genotypes was calculated in controls by a goodness-of-fit chi-squared test. Logistic regression analysis was used to estimate the effect of PSE and genotype frequencies on ADHD susceptibility by computing odds ratios (ORs) and 95% confidence intervals (95% CIs). Four inheritance models (codominant, dominant, additive and recessive models) were estimated for each SNP. Meanwhile, the statistical power to detect the effects of the SNPs was calculated by Power v3.0.0^48,49^, and for SNPs with MAF of 0.11(rs6354), 0.43(rs1124491), and 0.44(rs553668), for example, we calculated that the power for our sample size to detect an OR of 1.50 was 43.6, 74.1 and 74.8%, respectively.

The Multifactor Dimensionality Reduction (MDR) 2.0 beta 8.1 program (https://sourceforge.net/projects/mdr/) was used to assess the gene-environment interactions between risk genes and the environmental risks. The details analytic protocol was described in the previous study^50,51^. Furthermore, multiplicative and additive interactions were computed. Multiplicative interaction was evaluated by the interaction term of logistic regression model. Synergy index (SI), attributable proportion due to interaction (AP), and relative excess risk due to interaction (RERI) and their corresponding 95% CIs were calculated by additive interaction analysis, as suggested by Tomas Andersson^52^. If there is no additive interaction, RERI and AP are equal to 0, or SI are equal to 1. The multiplicative and additive interaction analyses were adjusted for age of the child, gender, pregnancy age of mother, and postnatal smoking exposure. Since a higher proportion of males and younger ADHD children(<9 years old) was noted in our study.

All analysis were performed using SPSS version 22.0 software (SPSS Inc., Chicago, Illinois, USA). All tests were two-tailed, and statistical significance was defined at *P* < 0.05.

## Supplementary information

supplementary table
