# Supplementary material for: Prenatal Tobacco Exposure Modulated the Association of Genetic variants with Diagnosed ADHD and its symptom domain in children: A Community Based Case–Control Study
Source: Sci Rep. 2019 Mar 12;9:4274. doi: 10.1038/s41598-019-40850-w (PMC6414688; doi:10.1038/s41598-019-40850-w)
Supplement: Supplementary file 1 — supplementary table [file 41598_2019_40850_MOESM1_ESM.pdf]

Title page

Prenatal Tobacco Exposure Modulated the Association of Genetic variants with Diagnosed ADHD and its symptom domain in children: A Community Based Case–Control Study

Authors: Yanni Wang[1], Dan Hu[2], Wenjing Chen[1], Hongli Xue[3], Yukai Du[1]*

Author Affiliations:

[1]Department of Maternal and Child Health, School of Public Health, Tongji Medical College, Huazhong University of Science and Technology, Wuhan, Hubei, P.R. China.

[2]Department of Child Health Care, Hospital of Maternal and Child Health of Dalian, Dalian, Liaoning, P.R. China.

[3] Department of Maternal, Child and Adolescent Health, School of Public Health, Lanzhou University, Lanzhou, Gansu, P.R. China.

Supplementary

Table S1 Association between individual SNP and ADHD risk

Model	genotype	Controls, N(%)	ADHD, N(%)	OR (95% CI)*	*P**	P^{FDR}
SLC6A3 rs1042129 (N=398)						
Codominant	GG	173 (74.6)	118 (71.1)	1.00	0.509	0.735
	AG	55 (23.7)	43 (25.9)	1.15 (0.72-1.82)		
	AA	4 (1.7)	5 (3.0)	1.83 (0.48-6.97)		
Dominant	GG	173 (74.6)	118 (71.1)	1.00	0.353	
	AG/AA	59 (25.4)	48 (28.9)	1.19 (0.76-1.87)		
Recessive	GG/AG	228 (98.3)	161 (97.0)	1.00	0.371	
	AA	4 (1.7)	5 (3.0)	1.86 (0.48-7.17)		
Additive	---	---	---	1.21 (0.81-1.79)	0.269	0.499
SLC6A3 rs2550948(N=393)						
Codominant	GG	169 (74.1)	117 (70.9)	1.00	0.781	0.846
	AG	52 (22.8)	42 (25.4)	1.17 (0.72-1.89)		
	AA	7 (3.1)	6 (3.6)	1.24 (0.40-3.84)		
Dominant	GG	169 (74.1)	117 (70.9)	1.00	0.481	
	AG/AA	59 (25.9)	48 (29.1)	1.18 (0.75-1.86)		
Recessive	GG/AG	221 (96.9)	159 (96.4)	1.00	0.762	
	AA	7 (3.1)	6 (3.6)	1.19 (0.39-3.68)		
Additive	---	---	---	1.15 (0.78-1.68)	0.491	0.580
SLC6A3 rs3756450(N=393)						
Codominant	TT	79 (34.6)	70 (42.4)	1.00	0.294	0.637
	CT	107 (46.9)	68 (41.2)	0.71 (0.45-1.11)		
	CC	42 (18.4)	27 (16.4)	0.73 (0.40-1.31)		
Dominant	TT	79 (34.6)	70 (42.4)	1.00	0.123	
	CT/CC	149 (65.3)	95 (57.6)	0.72 (0.47-1.09)		
Recessive	TT-CT	186 (81.6)	138 (83.6)	1.00	0.621	
	CT	107 (46.9)	68 (41.2)	0.78 (0.52-1.18)		
Additive	---	---	---	0.82 (0.62-1.10)	0.181	0.392
ADRA2A rs2484516(N=398)						
Codominant	CC	196 (84.1)	150 (89.3)	1.00	0.351	0.652
	CG	35 (15.0)	17 (10.1)	0.64 (0.34-1.19)		
	GG	2 (0.9)	1 (0.6)	0.77 (0.07-8.71)		
Dominant	CC	196 (84.1)	150 (89.3)	1.00	0.152	
	CG/GG	37 (15.9)	18 (10.7)	0.64 (0.35-1.19)		
Recessive	CC/CG	231 (99.1)	167 (99.4)	1.00	0.863	
	GG	2 (0.9)	1 (0.6)	0.81 (0.07-9.19)		
Additive	---	---	---	0.68 (0.38-1.20)	0.170	0.442
ADRA2A rs553668(N=385)						
Codominant	AA	84 (37.3)	40 (25.0)	1.00	**0.038**	0.247
	AG	115 (51.1)	93 (58.1)	**1.69(1.06-2.71)**		

	GG	26 (11.7)	27 (16.9)	**2.07 (1.05-4.06)**		
Dominant	AA	84 (37.3)	40 (25.0)	1.00	0.013	
	AG/GG	141 (62.8)	120 (75.0)	1.76(1.12-2.77)		
Recessive	AA/AG	199 (88.4)	133 (83.1)	1.00	0.140	
	GG	26 (11.7)	27 (16.9)	1.47(0.87-2.67)		
Additive	---	---	---	**1.49(1.08-2.06)**	**0.014**	0.182
ADRA2A rs1800544 (N=383)						
Codominant	CC	54 (24.0)	36 (22.6)	1.00	0.373	0.629
	CG	113 (50.2)	92 (57.9)	1.16 (0.67-1.89)		
	GG	58 (25.8)	31 (19.5)	0.74 (0.43-1.48)		
Dominant	AA	54 (24.0)	36 (22.6)	1.00	0.914	
	AG/GG	171 (76.0)	123 (77.4)	1.03 (0.68-1.61)		
Recessive	AA/AG	167 (74.2)	128 (80.5)	1.00	0.170	
	GG	58 (25.8)	31 (19.5)	0.76 (0.49-1.19)		
Additive	---	---	---	0.85 (0.76-1.23)	0.459	0.648
ADRA2A rs2484516 (N=384)						
Codominant	CC	158 (70.5)	118 (73.8)	1	0.592	0.590
	CG	62 (27.6)	38 (23.7)	0.92 (0.57-1.43)		
	GG	4 (1.8)	4 (2.5)	1.49 (0.31-5.11)		
Dominant	CC	158 (70.5)	118 (73.8)	1	0.510	
	CG/GG	66 (29.5)	42 (26.2)	0.89 (0.54-1.35)		
Recessive	CC/CG	220(98.2)	156(97.6)	1	0.490	
	GG	4 (1.8)	4 (2.5)	1.43 (0.42-4.98)		
Additive	---	---	---	1.64 (0.28-9.54)	0.598	0.742
ADRA2A rs521674 (N=395)						
Codominant	TT	122 (53.3)	89 (53.6)	1.00	0.096	0.450
	AT	94 (41)	65 (39.2)	1.78 (0.95-3.01)		
	AA	13 (5.7)	12 (7.2)	1.31 (0.55-3.31)		
Dominant	TT	122 (53.3)	89 (53.6)	1.00	0.510	
	AT/AA	107(46.7)	77(46.4)	1.35(0.92-1.98)		
Recessive	TT/AT	216(94.3)	154(92.8)	1	0.592	
	AA	13 (5.7)	12 (7.2)	1.48 (0.36-6.11)		
Additive	---	---	---	1.02(0.89-2.20)	0.109	0.356
DRD2 rs1124491(N=385)						
Codominant	GG	76 (34.2)	59 (36.2)	1.00	0.071	0.309
	AG	113 (50.9)	92 (56.4)	1.02(0.65-1.61)		
	AA	33 (14.9)	12 (7.4)	0.46(0.22-0.98)		
Dominant	GG	76 (34.2)	59 (36.2)	1.00	0.610	
	AG/AA	146 (65.8)	104 (63.8)	0.89(0.58-1.38)		
Recessive	GG/AG	189 (85.1)	151 (92.6)	1.00	**0.022**	
	AA	33 (14.9)	12 (7.4)	**0.45(0.23-0.92)**		
Additive	---	---	---	0.78(0.56-1.08)	0.130	0.426
ANKK1 rs1800497(N=390)						
Codominant	GG	81 (36.0)	55 (33.3)	1.00	0.141	0.489

	AG	112 (49.8)	96 (58.2)	1.26 (0.80-1.98)		
	AA	32 (14.2)	14 (8.5)	0.66 (0.32-1.35)		
Dominant	GG	81 (36.0)	55 (33.3)	1.00	0.599	
	AG/AA	144 (64.0)	110 (66.7)	1.12 (0.73-1.74)		
Recessive	GG/AG	193 (85.8)	151 (91.5)	1.00	0.091	
	AA	32 (14.2)	14 (8.5)	0.57 (0.29-1.11)		
Additive	---	---	---	0.93 (0.67-1.28)	0.642	0.795
DRD4 rs916457(N=321)						
Codominant	CC	138 (80.7)	123 (82.0)	1.00	0.910	0.910
	CT	30 (17.5)	25 (16.7)	0.93 (0.51-1.68)		
	TT	3 (1.8)	2 (1.3)	0.69 (0.11-4.53)		
Dominant	CC	138 (80.7)	123 (82.0)	1.00	0.741	
	CT/TT	33 (19.3)	27 (18.0)	0.91 (0.51-1.62)		
Recessive	CC/CT	168 (98.2)	148 (98.7)	1.00	0.741	
	TT	3 (1.8)	2 (1.3)	0.70 (0.11-4.58)		
Additive	---	---	---	0.90 (0.54-1.51)	0.692	0.692
DRD4 rs747302 (N=382)						
Codominant	CC	94 (42.7)	68 (41.9)	1.00	0.611	0.656
	CG	105 (47.7)	84 (51.9)	1.10 (0.73-1.68)		
	GG	21 (9.6)	10 (6.2)	0.77 (0.38-1.66)		
Dominant	CC	94 (42.7)	68 (41.9)	1.00	0.866	
	CG/GG	126 (57.3)	95 (58.1)	1.06 (0.66-1.69)		
Recessive	CC/CG	199 (90.5)	152 (93.8)	1.00	0.363	
	GG	21 (9.6)	10 (6.2)	0.71 (0.38-1.58)		
Additive	---	---	---	0.97 (0.64-1.37)	0.790	0.950
DRD4 rs1800955 (N=381)						
Codominant	TT	88 (41.1)	57 (34.1)	1		
	CT	108 (50.5)	92 (55.1)	1.84 (0.96-3.21)	0.089	0.347
	CC	18 (8.4)	18 (10.8)	1.63 (0.75-2.21)		
Dominant	TT	88 (41.1)	57 (34.1)	1		
	CT -CC	126 (58.9)	110 (65.9)	1.34 (0.88-2.05)	0.203	
Recessive	CC/CT	196(91.6)	149(89.2)	1		
	TT	18 (8.4)	18 (10.8)	1.44 (0.54-2.67)	0.402	
Additive	---	---	---	1.66 (0.96-2.89)	0.349	0.689
DRD4 rs3758653 (N=396)						
Codominant	TT	123 (54.0)	86 (51.2)	1	0.803	0.982
	CT	91 (40.0)	74 (44.0)	1.21 (0.83-1.81)		
	CC	14 (6.0)	8 (4.8)	0.91(0.65-1.26)		
Dominant	TT	123 (54.0)	86 (51.2)	1		
	CT/CC	105 (46.0)	82 (48.8)	1.05 (0.70-1.58)	0.801	
Recessive	TT/CT	214(94.0)	160(95.2)	1		
	CC	14 (6.0)	8 (4.8)	0.89(0.87-1.81)		
Additive	---	---	---	1.06 (0.77-1.48)	0.785	0.895
SLC6A4 rs6354(N=397)						

Codominant	TT	186 (80.9)	118 (70.7)	1.00	**0.001**	**0.019**
	GT	39 (17.0)	49 (29.3)	1.97(1.21-3.21)		
	GG	5 (2.2)	0 (0.0)	---		
Dominant	TT	186 (80.9)	118 (70.7)	1.00	0.025	
	GT/GG	44 (19.1)	49 (29.3)	1.73(1.07-2.78)		
Recessive	TT/GT	225 (97.8)	167 (100.0)		**0.013**	
	GG	5 (2.2)	0 (0.0)	---		
Additive	---	---	---	1.42(0.92-2.21)	0.120	0.349
SNAP25 rs362986(N=398)						
Codominant	T/T	197 (85.3)	147 (88.0)	1.00	0.720	0.859
	C/T	31 (13.4)	19 (11.4)	0.87 (0.47-1.62)		
	C/C	3 (1.3)	1 (0.6)	0.47 (0.05-4.60)		
Dominant	T/T	197 (85.3)	147 (88)	1.00	0.551	
	C/T-C/C	34 (14.7)	20 (12)	0.83 (0.46-1.52)		
Recessive	T/T-C/T	228 (98.7)	166 (99.4)	1.00	0.501	
	C/C	3 (1.3)	1 (0.6)	0.48 (0.05-4.66)		
Additive	---	---	---	0.82 (0.48-1.42)	0.481	0.625
SNAP25 rs362986(N=395)						
Codominant	A/A	129 (56.1)	98 (59.4)	1.00	0.562	0.738
	C/A	90 (39.1)	62 (37.6)	0.92 (0.60-1.40)		
	C/C	11 (4.8)	5 (3.0)	0.56 (0.19-1.68)		
Dominant	A/A	129 (56.1)	98 (59.4)	1.00	0.531	
	C/A-C/C	101 (43.9)	67 (40.6)	0.88 (0.58-1.32)		
Recessive	A/A-C/A	219 (95.2)	160 (97)	1.00	0.311	
	C/C	11 (4.8)	5 (3.0)	0.58 (0.19-1.72)		
Additive	---	---	---	0.85 (0.60-1.22)	0.381	0.679
SNAP25 rs3746544(N=391)						
Codominant	T/T	138 (60.5)	102 (62.6)	1.00	0.478	0.779
	G/T	79 (34.6)	57 (35.0)	0.96 (0.62-1.48)		
	G/G	11 (4.8)	4 (2.5)	0.50 (0.15-1.62)		
Dominant	T/T	138 (60.5)	102 (62.6)	1.00	0.631	
	G/T-G/G	90 (39.5)	61 (37.4)	0.90 (0.59-1.37)		
Recessive	T/T-G/T	217 (95.2)	159 (97.5)	1.00	0.232	
	G/G	11 (4.8)	4 (2.5)	0.50 (0.16-1.63)		
Additive	---	---	---	0.86 (0.60-1.24)	0.423	0.645
SNAP25 rs6077690 (N=392)						
Codominant	A/A	68 (29.8)	42 (25.6)	1.00	0.262	0.681
	A/T	123 (54.0)	87 (53.0)	1.21 (0.74-1.95)		
	T/T	37 (16.2)	35 (21.3)	1.67 (0.91-3.08)		
Dominant	A/A	68 (29.8)	42 (25.6)	1.00	0.252	
	A/T-T/T	160 (70.2)	122 (74.4)	1.31 (0.83-2.08)		
Recessive	A/A-A/T	191 (83.8)	129 (78.7)	1.00	0.142	
	T/T	37 (16.2)	35 (21.3)	1.48 (0.88-2.49)		
Additive	---	---	---	1.28 (0.95-1.74)	0.111	0.721

*：The ORs and 95%CIs were calculated by univariate logistic regression analysis controlling for age and gender, under assumption that variant alleles were risk alleles

P^{FDR}：The *P*-values were adjusted by False Discovery Rate (Benjamini–Hochberg method) for multiple tests within the logistic regression analysis

Table S2 The association between *ADRA2A* gene(rs553668) and child ADHD and symptom domains

Prenatal smoking exposure	SNP	Cases(N)	Controls(N)	OR(95%CI)*	*P*-value*
All ADHD					
No exposure	AA	26	61	1	
Yes exposure	AA	14	23	1.36(0.58-3.22)	0.485
No exposure	AG+GG	76	115	1.65(0.93-2.90)	0.084
Yes exposure	AG+GG	44	26	3.54(1.73-7.25)	0.001
Inattention					
No exposure	AA	23	61	1	
Yes exposure	AA	12	23	1.25 (0.51-3.05)	0.628
No exposure	AG+GG	71	115	1.68(0.94-3.01)	0.082
Yes exposure	AG+GG	41	26	3.53(1.70-7.37)	0.001
Hyperactivity/impulsivity					
No exposure	AA	15	61	1	
Yes exposure	AA	8	23	1.63 (0.56-4.710)	0.369
No exposure	AG+GG	33	115	1.29 (0.62-2.69)	0.490
Yes exposure	AG+GG	25	26	4.17 (1.71-10.17)	0.002
ODD					
No exposure	AA	7	60	1	
Yes exposure	AA	4	23	1.82 (0.43-7.67)	0.417
No exposure	AG+GG	14	113	1.34 (0.48-3.73)	0.580
Yes exposure	AG+GG	14	25	5.05 (1.58-16.14)	0.006

*: The ORs and 95%CIs were adjusted by paternal and maternal educational level, pregnancy age of mother, family history of nervous system diseases, low birth weight, preterm birth, blood lead and postnatal tobacco smoke exposure.

Table S3 The association between *DRD2* gene(rs1124491) and child ADHD and symptom domains

Prenatal smoking exposure	SNP	Cases(N)	Controls(N)	OR(95%CI)*	*P*-value*
All ADHD					
No exposure	AA	7	27	1	
Yes exposure	AA	99	145	5.34(1.64-24.46)	0.031
No exposure	AG+GG	6	6	3.99(1.46-10.89)	0.007
Yes exposure	AG+GG	52	44	6.04(2.07-17.65)	0.001
Inattention					
No exposure	AA	6	27	1	
Yes exposure	AA	90	145	5.16 (1.13-23.62)	0.034
No exposure	AG+GG	6	6	3.70 (1.35-10.12)	0.011
Yes exposure	AG+GG	47	44	5.34(1.82-15.66)	0.002
Hyperactivity/impulsivity					
No exposure	AA	4	27	1	
Yes exposure	AA	47	145	4.93(0.89-27.50)	0.069
No exposure	AG+GG	4	6	2.23(0.73-6.85)	0.159
Yes exposure	AG+GG	30	44	4.91(1.46-16.50)	0.010
ODD					
No exposure	AA	1	27	1	
Yes exposure	AA	19	145	9.87(1.58-49.74)	0.021
No exposure	AG+GG	3	6	4.23(0.53-33.71)	0.173
Yes exposure	AG+GG	16	44	10.58(1.26-49.23)	0.030

*: The ORs and 95%CIs were adjusted by paternal and maternal educational level, pregnancy age of mother, family history of nervous system diseases, low birth weight, preterm birth, blood lead and postnatal tobacco smoke exposure.

Table S4 The association between *SLC6A4* gene(rs6354) and child ADHD and symptom domains

Prenatal smoking exposure	SNP	Cases(N)	Controls(N)	OR(95%CI)*	*P*-value*
All ADHD					
No exposure	TT	74	147	1	
Yes exposure	TT	44	39	2.13(1.20-3.78)	0.010
No exposure	TG+GG	34	32	1.97(1.10-3.52)	0.022
Yes exposure	TG+GG	15	12	1.79(0.75-4.28)	0.190
Inattention					
No exposure	TT	69	147	1	
Yes exposure	TT	42	39	2.01(1.19-3.57)	0.011
No exposure	TG+GG	30	32	1.92(1.06-3.49)	0.032
Yes exposure	TG+GG	12	12	2.13(0.97-4.98)	0.081
Hyperactivity/impulsivity					
No exposure	TT	36	147	1	
Yes exposure	TT	24	39	2.70(1.29-5.65)	0.008
No exposure	TG+GG	16	32	2.03(0.97-4.25)	0.060
Yes exposure	TG+GG	10	12	2.92(1.10-7.75)	0.031
ODD					
No exposure	TT	13	147		
Yes exposure	TT	13	39	3.69(1.39-9.78)	0.009
No exposure	TG+GG	8	32	2.84(1.04-7.76)	0.042
Yes exposure	TG+GG	6	12	4.41(1.32-14.78)	0.016

*: The ORs and 95%CIs were adjusted by paternal and maternal educational level, pregnancy age of mother, family history of nervous system diseases, low birth weight, preterm birth, blood lead and postnatal tobacco smoke exposure.
